# Estrogenic and mutagenic activities of *Crotalaria pallida* measured by recombinant yeast assay and Ames test

**DOI:** 10.1186/1472-6882-13-216

**Published:** 2013-09-04

**Authors:** Paula Karina Boldrin, Flávia Aparecida Resende, Ana Paula Oliveira Höhne, Mariana Santoro de Camargo, Lívia Greghi Espanha, Catarine Haidê Nogueira, Maria do Socorro F Melo, Wagner Vilegas, Eliana Aparecida Varanda

**Affiliations:** 1 Department of Biological Sciences, Faculty of Pharmaceutical Sciences of Araraquara, UNESP- São Paulo State University, Rodovia Araraquara-Jaú, Km 1, 14801-902 Araraquara, São Paulo, Brazil; 2 Chemical Institute of Araraquara, UNESP-São Paulo State University, c.p. 355, 14800-900 Araraquara, São Paulo, Brazil

**Keywords:** *Crotalaria pallida*, Mutagenicity, Ames test, Estrogenicity, Recombinant yeast assay (RYA)

## Abstract

**Background:**

*Crotalaria pallida* Ailton is a plant belonging to the Fabaceae family, popularly known as “rattle or rattlesnake” and used in traditional medicine to treat swelling of the joints and as a vermifuge. Previous pharmacological studies have also reported anti-inflammatory, antimicrobial and antifungal activities. Nevertheless, scientific information regarding this species is scarce, and there are no reports related to its possible estrogenic and mutagenic effects. Thus, the purpose of the present study was to investigate the estrogenic potential of *C. pallida* leaves by means of the Recombinant Yeast Assay (RYA), seeking an alternative for estrogen replacement therapy during menopause; and to reflect on the safe use of natural products to assess the mutagenic activity of the crude extract from *C. pallida* leaves, the dichloromethane fraction and stigmasterol by means of the Ames test.

**Methods:**

The recombinant yeast assay with the strain BY4741 of *Saccharomyces cerevisiae,* was performed with the ethanolic extract, dichloromethane fraction and stigmasterol isolated from the leaves of *C. pallida*. Mutagenic activity was evaluated by the *Salmonella*/microsome assay (Ames test), using the *Salmonella typhimurium* tester strains TA100, TA98, TA97 and TA102, with (+S9) and without (-S9) metabolization, by the preincubation method.

**Results:**

All samples showed estrogenic activity, mainly stigmasterol. The ethanolic extract from *C. pallida* leaves showed mutagenic activity in the TA98 strain (-S9), whereas dichloromethane fraction and stigmasterol were found devoid of activity.

**Conclusion:**

Considering the excellent estrogenic activity performed by stigmasterol in the RYA associated with the absence of mutagenic activity when evaluated by the Ames test, stigmasterol becomes a strong candidate to be used in hormone replacement therapy during menopause.

## Background

Medicinal plants have been traditionally used worldwide for the treatment of various human diseases. They have proved to be abundant sources of biologically active compounds, many of which have been used as lead compounds to develop new pharmaceuticals [1].

*Crotalaria pallida* Ailton is a species that belongs to the Fabaceae family, popularly known as “rattle or rattlesnake” due to the sound of their fruits when dry [2]. This species is used in traditional medicine, having its roots used to treat swelling of the joints, and its leaves as vermifuge [3]. Pharmacological studies have demonstrated it also presents anti-inflammatory, antimicrobial and antifungal functions [4-8]. However, there are no studies verifying the estrogenic potential of *C. pallida.*

One of the current interests, in the pharmacological area, is to find compounds with estrogenic activity to replace estrogen in hormone replacement therapy (HRT) during menopause, without the undesirable effects of estrogen, such as the elevation of breast cancer incidence [9,10]. For this reason, the development of safer and more effective drugs for menopause treatment is an urgent priority. Plants appear as a therapeutic option, since some of their constituents have the ability to bind to estrogen receptors and to act in preventing the discomfort caused by the hormonal imbalance associated with menopause. These constituents are also related to the reduction of cancer incidence, particularly estrogen-dependent cancers, such as breast cancer, cardiovascular diseases and osteoporosis [11-14].

Seeking to find options for drug development, the estrogenic potential of *C. pallida* leaves was investigated using the RYA (Recombinant Yeast Assay), in which recombinant yeast is utilized as an experimental model and the transcription of a reporter gene depends on the presence in the midst of compounds capable of binding to the estrogen receptor [15].

In the light of finding a good candidate for HRT and given that natural products are promising sources of novel potentially therapeutic agents, another purpose of the present study was to investigate the mutagenic activities of *C. pallida* by the Ames test, in order to contribute to a toxicological evaluation of the candidate samples to be employed in HRT.

## Methods

### Chemicals

Dimethylsulfoxide (DMSO), nicotinamide adenine dinucleotide phosphate sodium salt (NADP), D-glucose-6-phosphate disodium salt, magnesium chloride, L-histidine monohydrate, D-biotin, sodium azide (SAZ), 2-anthramine (2-AA), 4-nitro-*o*-phenylenediamine (NOPD), mitomycin C (MMC), 2-aminofluorene (2-AF), 17-β-estradiol, Triton X-100, SDS 10%, 2-mercaptoethanol and 4-methylumbelliferyl β-D-galactoside were purchased from Sigma Chemical Co (St. Louis, USA). Oxoid Nutrient Broth No. 2 (Oxoid, England) and Difco Bacto Agar (Difco, USA) were used as bacterial media.

### Plant material

Leaves of *C. pallida* Aiton were collected in Poços de Caldas, Minas Gerais state, Brazil, and authenticated by Prof. Dr. Luis Victor Silva Sacramento. The voucher of *C. pallida* (BOTU 28656) was deposited at the Herbarium of the Botanical Institute of São Paulo State University (UNESP), Botucatu, São Paulo.

### Extraction and isolation

The leaves collected were dried in an oven with air circulation at 40°C. The samples were weighed for three days daily until constant weight. The material was pulverized and then moistened with 70% ethanol. This material was then accommodated in percolator and submitted to leaching process with 70% ethanol. The extracts were monitored using TLC-silica gel 60-F_254_ precoated Al sheets, using chloroform: methanol: *n*-propanol:water (5:6:1:4, v:v) as developing solvent, visualized using UV (254 and 366 nm) and anisaldehyde sulphuric acid spray [16], until total depletion of the drug. Following, the extract was filtered with pleated filter paper and concentrated under reduced pressure (40ºC). After lyophilization, the 70% hydroalcoholic extract was obtained. The chromatographic determination of the EtOH 70% extract was performed by HPLC-DAD (HPLC-PDA, Jasco ^®^, Column Phenomenex Synergi Hydro ^®^ RP-18 250 × 4.6 mm id; 4 mm, 1.0 ml min-1, FM: A = H_2_O with 0.1% formic acid, B = acetonitrile, linear gradient 0-100% B 60 minutes). The chromatographic profile obtained from the extract of the leaves was assessed in a chromatographic comparison of the signals observed. Their absorption spectra (220–400 nm) allowed us to observe the presence of flavonoids, mainly derivatives of apigenin and luteolin (flavones) (λ = 254 nm) and phenolic acids and derivatives (λ = 254 nm) [17].

In order to obtain the dichloromethane fraction by partition, chromatography was performed (liquid-liquid extraction - LLE) with dichloromethane (DCM) and methanol:water (2:8 v/v) (3x), yielding two fractions with different polarities: a dichloromethane fraction (DCM-Fr) and an aqueous fraction (Aq-Fr). Each of the fractions obtained from the LLE rotary evaporator were dried in order to remove the solvent and then frozen so as to have their mass determined. The fractions obtained from the LLE were subjected to a new fractioning using two techniques: the first dichloromethane fraction (DCM-Fr) was submitted to chromatography using an open silica gel column as the stationary phase and hexane mobile phase. The second technique was applied to the Aq-Fr in Medium Pressure Liquid Chromatography (MPLC) column using packed with C_18_ in the reverse silica and methanol:water solvent systems. The fractions obtained in the procedure above were analyzed by HPLC-PDA in order to obtain a preliminary view of the substances. Comparing the chromatograms obtained from the MPLC fractions, the majority of the substances absorbing UV belong to the class of flavones.

Chromatography by MPLC did not result in the separation of the pure substance, making a further step of purification necessary, using a preparative HPLC-PDA in order to isolate the majority of substances. The chromatographic analysis of the dichloromethane fraction allowed us to infer that the Fr 23–33 fraction was practically pure. The isolated substance was called F2. The 1H NMR spectrum showed characteristic signals of hydrogen steroid [18]. A comparison of the NMR chemical shifts of 1 H NMR substance alone, with the records in the literature [18] allowed us to identify it as stigmasterol.

### Recombinant yeast assay (RYA)

RYA was performed essentially as in Garcia-Reyero et al. [15]. Briefly, the yeast strain BY4741 (MATa ura3Δ0 leu2Δ0 his3Δ1 met15Δ0) (EUROSCARF, Frankfurt, Germany), which was kindly provided by Dr. Benjamin Piña (CSIC, Barcelona, Spain), was transformed together with plasmids pH5HE0 and pVitBX2 [15].

The expression plasmid pH5HE0 contained the human estrogen hormone receptor gene HE0 [19], cloned into the constitutive yeast expression vector pAAH5 [20]. The reporter plasmid pVITB2x contained two copies of the pseudo-palindromic responsive estrogen element from the *Xenopus laevis* vitellogenin B1 gene (5’-AGTCACTGTGACC-3’), inserted into the unique KpnI site of pSFLΔ-178 K [21].

Transformed clones were first grown in 3 mL of rich complete medium at 30°C. Following, they were grown overnight in a minimal medium. The final culture was adjusted to an optical density (OD) of 0.1 at 600 nm and distributed in the wells of a siliconized 96-well polypropylene microliter plate (NUNC™, U96 PP 0.5 mL), at 90 μL in the first row. Aliquots of 10 μL of the crude extract, dichloromethane fraction and stigmasterol at initial concentrations of 0.01, 0.03 and 0.15 × 10^-6^ g/mL, respectively, were dispensed into wells in the first row and serial dilutions were prepared along the plate, containing the samples with dilution factors of 1:10, 1:30, 1:90, 1:270 and 1:810. All the used concentrations are determinate in previous experiments of toxicity.

A positive control was made by adding 17-β-estradiol at a final concentration of 10 nM. Moreover, we included a toxicity control by adding 10 nM of 17-β-estradiol to a sample with a dilution factor of 1:30, and 10% DMSO as negative control.

Plates were incubated for 6 h at 30°C under 120 rpm. After incubation, 50 μL of Y-PERTM (PIERCE™, Rockford, IL, USA) were added to each well and incubated at 30°C for further 30 min. Afterwards, 50 μL of assay buffer were added to the lysed cells. The assay buffer was prepared by mixing 100 mL Z-buffer, 1 mL Triton X-100 (Sigma), 1 mL SDS 10%, 70 μL 2-mercaptoethanol (Fluka) and 21 mg of 4-methylumbelliferyl β-D-galactoside (Sigma). The Z-Buffer is a combination of: 60 mM Na_2_HPO_4_, 40 mM NaH_2_PO_4_, 10 mM KCl and 1 mM MgSO_4_, pH 7.0.

After centrifugation, plates were read in a spectrofluorometer (Synergy H1, Biotek), at 355 nm excitation and 460 nm emission wavelengths. Fluorescence was recorded for 20 min (one measurement per min); β-galactosidase activity was calculated as the rate of increase of fluorescence (in arbitrary units). RYA does not provide a direct measurement of the molar (or mass) concentration of endocrine disruptors, but of their estrogenic activity. For simplification, results were calculated as estradiol equivalents (EEQ), defined as the amount of estradiol that should be present to account for the observed response in a given sample. These equivalents were calculated from the lowest dilution in which the β-galactosidase activity was indistinguishable from that of the control (only vehicle).

To translate results from serial dilutions to EEQ, we assumed that hormonal dose–response curves follow a sigmoidal function,

$$\frac{R‒R0}{\text{Rmax}‒R0}=\frac{1}{1+\frac{\mathrm{Kd}}{\left[L\right]}}$$

In which R0, R, and Rmax represent *β*-galactosidase units obtained without ligand (or extract) addition, at a given ligand concentration [L], and at a saturating ligand concentration, respectively. Kd represents the dissociation constant of the ligand–hormone complex; its value coincides with EC_50_, the ligand concentration giving 50% of the maximal response. For extract serial dilutions, plotting dilution factors versus relative response followed an inverse sigmoidal function, in which the apparent EC_50_ correspond to the dilution (actual or theoretical) giving 50% the response for 10 nM estradiol. Apparent EC_50_ values for each sample (a minimum of two replicas with at least four points each) were calculated using standard non-linear regression methods. These values were converted to EEQ by assuming they correspond to the EC_50_ of estradiol [22], 7.29 × 10^-9^ g/mL in our assay. Three independent experiments were done with the compounds, all of them in triplicate.

### *Salmonella*/microsome assay

Mutagenic activity was evaluated by the *Salmonella*/microsome assay, using the *Salmonella typhimurium* tester strains TA98, TA100, TA97a and TA102, which were kindly provided by Dr. B.N. Ames (Berkeley, CA, USA), with (+S9) and without (-S9) metabolization, using the preincubation method [23]. The strains were grown overnight from frozen cultures for 12–14 h in Oxoid Nutrient Broth No. 2. The metabolic activation mixture (S9 fraction), prepared from livers of Sprague–Dawley rats treated with the polychlorinated biphenyl mixture Aroclor 1254 (500 mg/kg), was purchased from Molecular Toxicology Inc. (Boone, NC, USA) and freshly prepared before each test. The metabolic activation system consisted of 4% S9 fraction, 1% 0.4 M MgCl2, 1% 1.65 M KCl, 0.5% 1 M D-glucose-6-phosphate disodium and 4% 0.1 M NADP, 50% 0.2 M phosphate buffer and 39.5% sterile distilled water [23]. For the determination of the mutagenic activity, five different concentrations of each sample (0.7 to 18.9 mg/plate for extract, 0.3 to 3.4 mg/plate for dichloromethane fraction and 0.02 to 0.5 mg/plate for stigmasterol), diluted in DMSO, were assayed. The mutagenic activity of the dichloromethane fraction and of the stigmasterol was only evaluated in the TA98 strain, because of the small amount of material available for the study and based in the fact that initially mutagenic activity was only observed for TA98. The concentrations of the samples were selected based on a preliminary toxicity test. In all subsequent assays, the upper limit of the dose range tested was either the highest non-toxic dose or the lowest toxic dose determined in this preliminary assay. Toxicity was detected either as a reduction in the number of histidine revertants (His+), or as a thinning of the auxotrophic background (*i.e.*, background lawn).

The various concentrations of extract, fraction and isolated substance to be tested were added to 0.5 mL of 0.2 M phosphate buffer or to 0.5 mL of 4% S9 mixture, with 0.1 mL of bacterial culture and then incubated at 37°C for 20–30 min. Following, 2 mL of top agar were added and the mixture poured on to a plate containing minimal agar.

The plates were incubated at 37°C for 48 h and the His+ revertant colonies were counted manually. All experiments were analyzed in triplicate. The results were analyzed with the statistical software package Salanal 1.0 (U.S. Environmental Protection Agency, Monitoring Systems Laboratory, Las Vegas, NV, from the Research Triangle Institute, RTP, NC, USA), adopting the model of Bernstein et al. [24]. The data (revertants/plate) were assessed by means of the analysis of variance (ANOVA), followed by linear regression. The mutagenicity ratio (MR) was also calculated for each concentration tested, this being the mean number of revertants per plate with the test compound divided by the mean number of revertants per plate with the negative (solvent) control. A test solution was considered mutagenic when a dose–response relationship was detected and a two-fold increase in the number of mutants (MR ≥ 2) was observed in at least one concentration [1]. The standard mutagens used as positive controls in experiments without the S9 mix were NOPD (10 μg/plate) for TA98 and TA97a, SAZ (1.25 μg/plate) for TA100 and MMC (0.5 μg/plate) for TA102. In experiments with S9 activation, 2-AA (1.25 μg/plate) was used with TA98, TA97a and TA100 and 2-AF (10 μg/plate) with TA102. DMSO served as negative (solvent) control.

## Results

### Estrogenicity assay (RYA)

The result of the estrogenic activity assessment, performed using the RYA, was given as equivalent to estradiol (EEQ) and EC_50_ (Table 1). The extract from *C. pallida* leaves obtained an EEQ of 14.3 nM in the RYA, whereas the fraction obtained 89.0 nM. The isolated substance obtained the highest value equivalent to estradiol, 122 nM. Regarding the EC_50_ values, stigmasterol proved to be the most potent sample, with EC_50_ values of 10.5 × 10^-7^ g/mL.

**Table 1:** **Estrogenic activity expressed through the values of estradiol equivalents (EEQ) ± standard deviation of the extracts and EC**_**50 **_**values of the ethanol extract of *C. pallida*, dichloromethane fraction and stigmasterol using a genetically modified strain of *S. cerevisae*** BY4741

**Samples**	**EEQ (nM) M ± SD**	**EC**_**50 **_**(g/mL)**
Ethanolic extract	14.3 ± 4.1	0.2
Dichloromethane fraction	89.0 ± 16.0	0.1
Stigmasterol	122.0 ± 11	10.5 × 10^-7^
17-β-estradiol	-	7.29 × 10^-9^

*EEQ*, Values of estradiol equivalents (EEQ); *M ± SD*, Mean and standard deviation; *EC*_*50*_, Concentration of sample equivalent to 50% of the activity.

**Figure 1:**
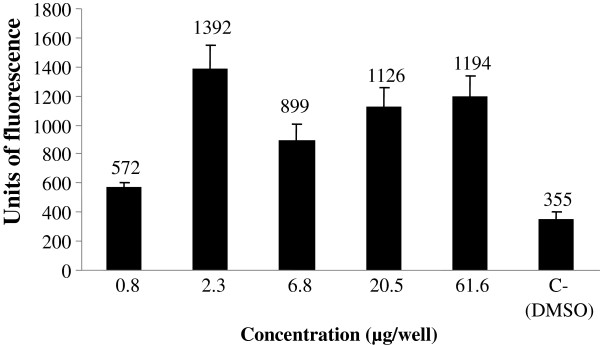
**Estrogenic response for extract of *C. pallida *leaves in the recombinant yeast assay.** Different concentrations of extract of *C. pallida* leaves (μg/well) were added to genetically engineered, estrogen- responsive yeast cells and incubated for 6 h. The β-galactosidase activities were calculated as fluorescence units (FU). Values are averages of three independent experiments; bars indicate value ranges. Negative control, DMSO, FU = 355 ± 21; positive control, 17-β-estradiol, FU = 9834 ± 985.

**Figure 2:**
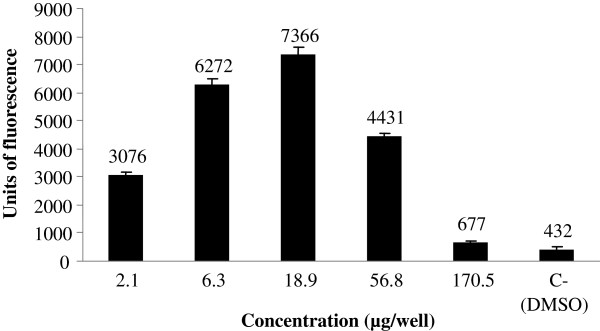
**Estrogenic response for extract dichloromethane fraction of *C. pallida*, in the recombinant yeast assay.** Different concentrations of dichloromethane fraction of *C. pallida*, (μg/well) were added to genetically engineered, estrogen- responsive yeast cells and incubated for 6 h. The β-galactosidase activities were calculated as fluorescence units (FU). Values are averages of three independent experiments; bars indicate value ranges. Negative control, DMSO, FU = 432 ± 34; positive control, 17-β-estradiol, FU = 11490 ± 496.

**Figure 3:**
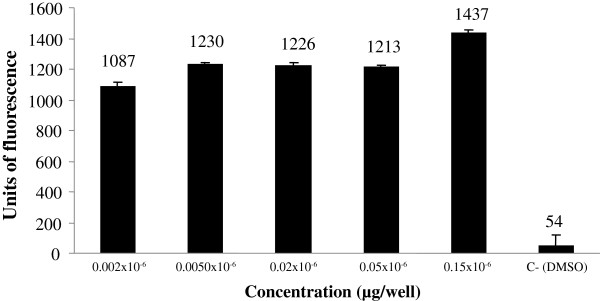
**Estrogenic response for stigmasterol, isolated from *C. pallida *leaves, in the recombinant yeast assay.** Different concentrations of stigmasterol, (μg/well) were added to genetically engineered, estrogen- responsive yeast cells and incubated for 6 h. The β-galactosidase activities were calculated as fluorescence units (FU). Values are averages of three independent experiments; bars indicate value ranges. Negative control, DMSO, FU = 54 ± 16; positive control, 17-β-estradiol, FU = 9678 ± 167.

Figure 1 shows the estrogenic activity, assessed by the β-galactosidase activity expressed in arbitrary units of fluorescence of five concentrations of the extract. It is possible to observe that the different concentrations tested presented significant enzymatic activation when compared to the negative control and the estrogenic activity of the extract starting from the second lowest concentration. The dichloromethane fraction (Figure 2) also generated an increase of fluorescence units, even at lower concentration that is similar to the second lowest concentration of the extract. The concentration of 18.9 μg/well provided the greatest estrogenic activity for this sample. Figure 3 shows the estrogenic activity, expressed in fluorescence units, for stigmasterol isolated from *C. pallida* leaves. The average fluorescence was compared to negative control. This sample generated a significant activation of the β-galactosidase enzyme, at all concentrations tested, and the units of fluorescence were greater than those found in the negative control.

### Mutagenicity assay (Ames test)

Table 2 shows the mean number of revertants/plate, the standard deviation (SD) and the MR after treatment with the five concentrations of crude extract, observed in *S. typhimurium* strains TA98, TA100, TA97a and TA102, in the presence (+S9) and absence (-S9) of metabolic activation. The mutagenic activity was considered negative for the strains TA97, TA102 and TA100 at all concentrations tested, both in the absence and in the presence of metabolism. However, in the absence of the external metabolizing system (-S9), the crude extract was mutagenic, with a mutagenic ratio higher than 2.0 at the concentrations of 9.0 and 13.5 mg/plate in the TA98 strain. Thus, the Ames test was applied to the fraction and to stigmasterol in the same conditions, using the TA98 strain, since the extract showed mutagenic activity for this strain.

**Table 2:** **Mutagenic activity expressed by the mean number of revertants/plate ± standard deviation and mutagenicity ratio (in brackets) of the ethanol extract of *C. pallida *leaves in *S. typhimurium *strains in the absence (-S9) and in the presence of metabolism (+S9)**

**Extract from *Crotalaria pallida *leaves**										
**Treatment**	**TA97a**		**TA102**		**Treatment**	**TA100**		**Treatment**	**TA98**	
**mg/plate**	**-S9**	**+S9**	**-S9**	**+S9**	**mg/plate**	**-S9**	**+S9**	**mg/plate**	**-S9**	**+S9**
DMSO	108 ± 4	140 ± 21	170 ± 6	196 ± 10	DMSO	110 ± 0,6	185 ± 6	DMSO	43 ± 2	20 ± 4
0.7	129 ± 14 (12)	177 ± 12 (1.3)	150 ± 24 (0.9)	224 ± 6 (1.1)	0.9	122 ± 2 (1.1)	237 ± 13 (1.3)	1.8	51 ± 0 (1.2)	18 ± 2 (0.9)
1.9	119 ± 4 (1.1)	164 ± 2 (1.2)	143 ± 5 (0.8)	251 ± 5 (1.3)	2.3	106 ± 7 (1.0)	295 ± 51 (1.1)	4.5	65 ± 8 (1.5)	18 ± 1 (0.9)
3.7	109 ± 5 (1.0)	141 ± 5 (1.0)	146 ± 6 (0.9)	275 ± 19 (1.4)	4.5	145 ± 5 (1.3)	203 ± 3 (1.1)	9.0	95 ± 13* (2.2)	17 ± 2 (0.9)
5.6	97 ± 3 (0.9)	134 ± 12 (1.0)	128 ± 5 (0.8)	265 ± 8 (1.4)	6.8	118 ± 6 (1.1)	222 ± 8 (1.2)	13.5	95 ± 22* (2.2)	21 ± 2 (1.0)
7.4	117 ± 6 (1.1)	174 ± 4 (1.2)	194 ± 5 (1.1)	219 ± 2 (1.1)	9.0	83 ± 5 (0.8)	167 ± 36 (0.9)	18.0	77 ±12 (1.8)	19 ± 1 (0.9)
Control +	1488 ± 18^a^	2253 ± 56^b^	1720 ± 26^c^	1168 ± 28^d^	Control +	1312 ± 60^e^	1195 ± 157^b^	Control +	1050 ± 39^a^	1063 ± 24^b^

*p < 0.05 (ANOVA); Negative Control: dimethyl sulfoxide (DMSO - 100 μL/plate); Positive Control (Control+); ^a^4-nitro-*o*-phenylenediamine (10.0 μg/plate); ^b^2-anthramine (1.25 μg/plate); ^c^mitomycin (0.5 μg/plate); ^d^2-aminofluorene (10.0 μg/plate); ^e^sodium azide (1.25 μg/plate).

As shown in Table 3, no mutagenic activity was observed for the dichloromethane fraction at any of the concentrations examined for TA98 strain. Stigmasterol was not found mutagenic for the TA98 strain in the presence and absence of metabolizing enzymes either. As shown in Table 4, no MR greater than 2 was observed.

**Table 3:** **Mutagenic activity expressed by the mean number of revertants/plate ± standard deviation and mutagenicity ratio (in brackets) of dichloromethane fraction of *C. pallida *in *S. typhimurium *strain (TA98) in the absence (-S9) and in the presence of metabolism (+S9)**

**Dichloromethane fraction of *C. pallida***		
**Treatment**	**TA98**	
**mg/plate**	**-S9**	**+S9**
DMSO	14 ± 3	27 ± 3
0.3	19 ± 2 (1.4)	29 ± 6 (1.1)
0.9	15 ± 2 (1.1)	27 ± 2 (1.0)
1.7	15 ± 2 (1.1)	29 ± 5 (1.1)
2.6	17 ± 3 (1.2)	33 ± 4 (1.2)
3.4	19 ± 4 (1.4)	31 ± 2 (1.1)
Control+	751 ± 96^a^	1088 ± 109^b^

Negative Control: dimethyl sulfoxide (DMSO - 100 μL/plate); Positive Control (Control+); ^a^4-nitro-*o*-phenylenediamine (10.0 μg/plate); ^b^2-anthramine (1.25 μg/plate).

**Table 4:** **Mutagenic activity expressed by the mean number of revertants/plate ± standard deviation and mutagenicity ratio (in brackets) of stigmasterol, isolated substance of dichloromethane fraction of *C. pallida *leaves in *S. typhimurium *strain (TA98) in the absence (-S9) and in the presence of metabolism (+S9)**

**Stigmasterol**		
**Treatment**	**TA 98**	
**mg/plate**	**- S9**	**+S9**
DMSO	16 ± 2	25 ± 2
0.02	16 ± 3 (1.0)	31 ± 4 (1.2)
0.04	17 ± 2 (1.1)	23 ± 4 (0.9)
0.09	15 ± 1 (0.9)	27 ± 2 (1.1)
0.18	17 ± 0 (1.1)	22 ± 1 (0.9)
0.5	19 ± 1 (1.2)	30 ± 5 (1.2)
Control +	815 ± 21^a^	1531 ± 36^b^

Negative Control: dimethyl sulfoxide (DMSO - 100 μL/plate); Positive Control (Control+); ^a^4-nitro-*o*-phenylenediamine (10.0 μg/plate); ^b^2-anthramine (1.25 μg/plate).

## Discussion

RYA was used to identify compounds with structurally similar estrogenic activity to the natural estrogen, 17-β-estradiol, and which can interact with the ER. This test utilizes an engineered yeast strain that harbors two foreign genetic elements: a vertebrate receptor, in this case, a human estrogen receptor (ER), and a reporter gene, whose expression is made dependent on the presence of estrogens and whose final product concentration is easy to measure [25].

The samples showed significant estrogenic activity when evaluated by RYA. The crude extract of *C. pallida* leaves showed an equivalent value to estradiol of 14.5 nM, which evidences that this extract has compounds with affinity for the ER present in yeast. The extract promoted a significant activation of the β-galactosidase enzyme, which could be observed due to the high fluorescence units. The same could be observed with the dichloromethane fraction, which presented an equivalent value to estradiol of 89.0 nM and promoted a high value of fluorescence units. Since the dichloromethane fraction of leaves from this plant stood out due to their estrogenic potential, there was a great interest in discovering the substance responsible for the activity that was so evident in the leaves.

The chromatographic profile of the leaves revealed the presence of flavones, for instance apigenin and luteolin derivates. Flavonoids have some structural similarities to the natural estrogen 17-β-estradiol, as well as to other steroid hormones and steroid hormone antagonists [26], and can interact with the ER and induce gene expression similar to that induced by estrogens, albeit at a lower affinity [27].

Regarding the structure of flavonoids and their possible estrogenic activity, Zand et al. [26] reported that hydroxyl at the 6, 7 or 4’ positions of the flavonoid provides greater estrogenic activity and more potent compounds with 2–4 hydroxyl groups, namely, at least one in the 7 position of the ring A and another in the 4’ position of ring B. The presence of these hydroxyl groups is essential for the estrogenic activity because they mimic the hydroxyl at the 3 and 17 position, found in the 17-β-estradiol molecule [28]. Furthermore, the presence of a double bond between carbons 2 and 3 of the ring C is essential for the estrogenic activity [26]. These structural features are exhibited by the apigenin and luteolin molecules, which allow a good interaction with ER.

However, derivatives of flavonoids, such as apigenin and luteolin, usually appear glycosylated [29], and this glycosylation can eliminate the free hydroxyl that is essential for the interaction with ERs or substituents may provide steric hindrance that also hinders the interaction with ERs. Furthermore, the ortho positioning between two hydroxyl groups may serve to reduce estrogenicity, due to the avoidance of direct interaction with ER [30], as observed with the luteolin molecule. Thus, these flavones were probably not responsible for the estrogenic activity performed by the extract.

In view of the promising results obtained in experiments with extract of leaves and considering that the extract is a complex mixture of several unknown organic compounds [1], the evaluation of isolated compounds is even more relevant. In this context, stigmasterol was isolated from the dichloromethane fraction of leaves and, following, its estrogenic activity assessed by RYA.

The results for stigmasterol in RYA showed that it is highly likely that this substance is responsible for the high estrogenicity found in leaves of *C. pallida*, since it was able to provide the highest result equivalent to estradiol (122.0 nM) and an important EC_50_ value of 10.5 × 10^-7^ g/mL.

Stigmasterol belongs to the class phytosteroids extracted from species of plants. Among the most common phytosteroids are β-sisterol, campesterol and stigmasterol. Chemically, they are alcohols consisting of 28 or 29 carbon atoms, similar to cholesterol [31].

The advantages of phytosteroids result from its biodegradability, the ability to affect numerous biological processes through membranes, to bind to specific hormone receptors and to be modified by synthetic methods [32-34].

**Figure 4:**
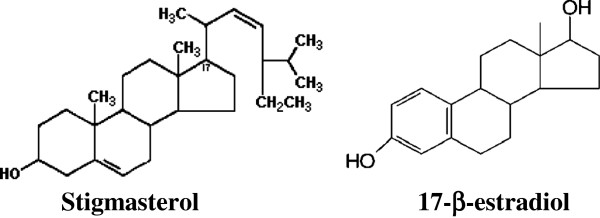
Structural similarity between stigmasterol and 17-β-estradiol.

The structural similarity between the molecule and stigmasterol from 17-β-estradiol (Figure 4) is remarkable, justifying the high estrogenic activity found in this sample by means of the RYA.

Since one of the purposes of this study was to search for compounds with estrogenic activity that could be used in hormone replacement therapy, the results obtained for the samples, mainly for stigmasterol, in the RYA, make these substances possible candidates.

Regarding the safe use of natural products, the mutagenic activity of the crude extract of *C. pallida* leaves, dichloromethane fraction and stigmasterol were assessed by the Ames test. Although the crude extract of *C. pallida* leaves showed good results when assessed as for its estrogenic activity, it proved to be mutagenic when evaluated by the Ames test. The extract was mutagenic for the TA98 strain, in the absence of metabolic activity. This allows us to state that this extract has direct mutagens acting as per the mechanism of *frameshift*.

In relation to the study of the mutagenic potential of flavonoids, Rietjens et al. [35] have described essential features in the flavonoid structure for the mutagenic activity to be present, such as: a free hydroxyl at the 3 position of the ring C, double bond between the 2 and 3 positions and the keto group at the 4 position. These characteristics are fundamental for the presence of mutagenic activity because they allow the hydroxyl at the 3 position to tautomerize molecule for molecule 3-keto. However, the apigenin and luteolin molecules do not have hydroxyl at the 3 position of the ring C, which may suggest that these substances are not responsible for the mutagenic activity found in the extract.

The literature reports that pyrrolizidine alkaloids are often found in *Crotalaria* species, including *C. pallida*[36,37]. Previous studies have demonstrated that alkaloids are able to induce chromosomal aberrations in the CHO (Chinese Hamster Ovary) [38] cell line, mutagenicity in Chinese hamster lung cells (CHL) [39], sister chromatid exchange in human peripheral lymphocytes [40] and increase the frequency of micronuclei and sister chromatid exchange in meristematic cells of *Allium cepa*[41]. The hepatotoxic activity of pyrrolizidine alkaloids probably results from the high reactivity of alkylating pyrrole derivatives, generated during enzymatic metabolism [42]. Both alkaloid molecules and metabolites are reactive alkylating agents capable of forming covalent bonds with stable macromolecules [43,44]. Due to the ability of pyrrolizidine alkaloids to interact with the genetic material, their involvement has been evidenced with mutagenicity and carcinogenesis [43].

In this study, the dichloromethane fraction and stigmasterol did not show mutagenic activity when evaluated by the Ames test in the TA98 strain. Considering the promising estrogenic activity of this isolated substance, a lack of mutagenic effect in bacterial systems is highly relevant.

Phytosterols such as stigmasterol are known as antioxidants [45], playing a role in the chemoprevention of DNA damage induced by oxidative radicals [46]. The exact mechanism by which phytosteroids offer protection against cancer is not understood, but there are theories proposing their effect on the structure of cell membranes, on the fluidity of cell membranes, on enzymes bound to membranes, on signal transduction pathways, on apoptosis, on membrane integrity, on the immune function, on the estrogenic properties on the tissue and on acids and neutral steroids in the colon [31].

Lim et al. [47] reported stigmasterol as a substance with high antimutagenic activity in *Gleditsia sinensis* Lam, Leguminosae, obtaining a reduction of 51.2% and 64.2% of mutagenicity against MNNG (N-methyl-N’-nitro-nitrosoguanidine) and 4-NQO (4-nitroquinoline-N-oxide) mutagens, respectively, by means of *in vitro* assay, referring to the application of stigmasterol as an anticancer agent, but emphasizing the need for *in vivo* studies. Substances with antimutagenic potential increase the efficiency of the repair mechanisms of mutations caused by mutagens or cause the inactivation of the mutagenic substance. As a result, these substances act as protective agents, reducing the frequency of DNA damage. In both cases, however, they act before the disordered multiplication of cells or tumor formation [48].

Considering the promising estrogenic activity and that stigmasterol might be successfully incorporated into pharmaceutical products, the absence of a mutagenic effect in the Ames assay is a positive step towards determining its safe use in hormone replacement therapy during menopause.

## Conclusions

In conclusion, this study evidenced that the crude extract of *C. pallida* leaves has an estrogenic effect. Nevertheless, the present results indicate that this extract should be used with caution because it might be mutagenic. Considering that medicinal herbs contain complex mixtures of thousands of components that can act alone or synergistically, the estrogenic activity of dichloromethane fraction and stigmasterol isolated from *C. pallida* leaves with absence of a mutagenic effect in the Ames assay is highly relevant, since these samples may be successfully incorporated into pharmaceutical products with an important role in hormone replacement therapy.

## Abbreviations

NOPD: 4- Nitro-*o*-phenylenediamine; SAZ: Sodium azide; MMC: Mitomycin C; 2-AA: 2-Anthramine; 2-AF: 2-Aminofluorene; DMSO: Dimethylsulfoxide; PBS: Phosphate buffer saline; +S9: With metabolization; –S9: Without metabolization; MR: Mutagenicity ratio.

## Competing interests

The authors declare that they have no competing interests.

## Authors’ contributions

PKB designed and performed the experiments, interpreted the results and drafted the manuscript. APSO, MSC and FAR designed and performed the Recombinant Yeast Assay. LGE and CHN participated in the experiments of the Ames test. MSFM and WV prepared the ethanol extract of *C. pallida* and dichloromethane fraction, and isolated the stigmasterol. FAR and EAV critically read the manuscript and participated in its revision. All authors have read and approved the final manuscript.

## Pre-publication history

The pre-publication history for this paper can be accessed here:

http://www.biomedcentral.com/1472-6882/13/216/prepub
